# Population heterogeneity and dynamics in starter culture and lag phase adaptation of the spoilage yeast *Zygosaccharomyces bailii* to weak acid preservatives

**DOI:** 10.1016/j.ijfoodmicro.2014.04.017

**Published:** 2014-07-02

**Authors:** Malcolm Stratford, Hazel Steels, Gerhard Nebe-von-Caron, Simon V. Avery, Michaela Novodvorska, David B. Archer

**Affiliations:** a School of Life Sciences, University of Nottingham, University Park, Nottingham, NG7 2RD, United Kingdom; b Mologic Ltd., Bedford Technology Park, Thurleigh, Bedford, MK44 2YP, United Kingdom

**Keywords:** Spoilage, Sorbic acid, Population diversity, Adaptation, Lag phase, Resistant sub-population

## Abstract

The food spoilage yeast *Zygosaccharomyces bailii* shows great resistance to weak-acid preservatives, including sorbic acid (2, 4-hexadienoic acid). That extreme resistance was shown to be due to population heterogeneity, with a small sub-population of cells resistant to a variety of weak acids, probably caused by a lower internal pH reducing the uptake of all weak acids. In the present paper, it was found that resistant cells were extremely rare in exponential cultures, but increased by up to 8000-fold in stationary phase. Inoculation of media containing sorbic acid with a population of *Z. bailii* cells gave rise to what appeared to be a prolonged lag phase, suggesting adaptation to the conditions before the cells entered the period of exponential growth. However, the apparent lag phase caused by sorbic acid was largely due to the time required for the resistant sub-population to grow to detectable levels. The slow growth rate of the sub-population was identical to that of the final total population. The non-resistant bulk population remained viable for 3 days but had lost viability by 6 days and, during that time, there was no indication of any development of resistance in the bulk population. The sub-population growing in sorbic acid showed very high population diversity in colony size and internal pH. After removal of sorbic acid, the population rapidly reverted back to the normal, largely non-resistant, population distribution. The data presented suggest that a reevaluation of the lag phase in microbial batch culture is required, at least for the resistance of *Z. bailii* to sorbic acid. Furthermore, the significance of phenotypic diversity and heterogeneity in microbial populations is discussed more broadly with potential relevance to bacterial “persisters”, natural selection and evolution.

## Highlights

• Extreme *Z. bailii* resistance to sorbic acid was due to population heterogeneity.
• There was a massive rise in resistant cells after exponential growth.
• Extended lag phase caused by weak acids was not an adaptive response.
• Prolonged lag phase was due to population replacement.
• Stress, starvation or weak acid, caused a rise in population diversity.

## 1 Introduction

Adaptation or adaptive response is often used to describe the adjustment of gene expression to enable growth in a stressed environment. As an example, when yeast cells are inoculated into a new medium, there is typically a brief delay (Brejning et al., 2005) until measurable growth is initiated, termed the lag phase (Lewis et al., 1993). When the conditions in the medium are stressful, the lag phase frequently becomes extended. Prolonged lag phases have been observed in response to a variety of stresses including ethanol (Vriesekoop and Pamment, 2005), cadmium (Minney and Quirk, 1985), CO_2_ (Liao et al., 2008), essential oils (Gutierrez et al., 2008) and weak acid preservatives (Neves et al., 1994; Stratford and Anslow, 1996), all of which have been shown to extend the lag phase for several days. It is widely assumed that during the prolonged lag phase, new gene expression patterns are induced to overcome the stress.

Alternatively, adaptation has been used to describe an increase in resistance, following a prior exposure to low levels of stress. For example, the elevated heat resistance of yeast following development of a “heat-shock response”, when cells are first warmed to a sub-lethal temperature, enables cells to survive an otherwise lethal heat shock (Ellis and Hemmingsen, 1989; Steels et al., 1994). Other studies have shown that bacteria suddenly exposed to acidic pH will rapidly die, whereas cultures acclimatised to mildly acidic conditions express an acid tolerance response (ATR) and subsequently survive better at low pH (Baik et al., 1996; Huhtanen, 1975).

In the food industry, early studies of spoilage of imported orange juice concentrates (Ingram, 1960; Ingram et al., 1956) described adaptation as enhanced resistance of yeasts to preservatives, being a result of previous exposure to preservatives at lower concentrations. It is generally envisaged that within factories, spillages of food products containing preservatives could allow growth of an adapted spoilage flora. Physiological tests on adaptation to benzoic acid (Warth, 1977, 1988) showed that the concentration required to inhibit the growth of yeast increased between 40 and 100% after prior growth in the preservative. Similarly, adaptation to acetic acid and propionic acid was reported by Malfeito Ferreira et al. (1997).

It has long been known that the morphology of fungal cells can vary considerably between cells with identical genomes. Dimorphism in yeasts is relatively common, perhaps best studied in *Candida albicans* in the transition between yeast and hyphal forms (Hornby et al., 2001). Other yeast or mould cells may be induced under certain environmental conditions to form spores, such as heat-resistant ascospores (Beuchat and Rice, 1979; Tournas, 1994). Such major physiological changes are promoted by substantial changes in patterns of gene expression. Yet when populations of morphologically similar cells are considered, it has historically been a common assumption that all genetically identical cells in a constant environment show identical patterns of gene expression. However, evidence has been accumulating of population heterogeneity within genetically homogeneous populations (Raser and O'Shea, 2004). In yeasts, survival of metal toxicity was limited to a small fraction of the population (Bishop et al., 2007; Smith et al., 2007), as has also been shown in regard to the preservative sorbic acid (Steels et al., 2000) and for survival of heat shock (Levy et al., 2012).

In a previous paper, it was shown that the spoilage yeast *Zygosaccharomyces bailii* was resistant to a variety of toxic weak acids, due to a long “tail” of increasingly resistant cells within the population (Stratford et al., 2013). The resistant sub-population exhibited cross-resistance to all other weak acids, showing that resistance was not dependent on the acid structure and implying a mechanism that lowered uptake of all acids. The theory that the resistant sub-population had a lower cytoplasmic pH, which reduced the quantity of weak acids accumulated, was proposed.

In the current paper, the presence of the resistant cell fractions within genetically-uniform populations of the spoilage yeast *Z. bailii* is examined. The population dynamics during the adaptation process and during loss of adaptation are measured, both in terms of events during lag phase, and changes in the concentrations of preservatives required to prevent growth.

## 2 Materials and methods

### 2.1 *Z. bailii* strain

The *Z. bailii* strain used in this study was NCYC 1766. This was selected as a typical strain of the 38 *Z. bailii* strains examined previously (Stratford et al., 2013). The yeast strain was stored in glycerol on ceramic beads at − 80 °C (Microbank™).

### 2.2 Growth media

The growth medium used in all experiments was YEPD; glucose 20 g/l, bacteriological peptone (Oxoid) 20 g/l, and yeast extract (Oxoid) 10 g/l adjusted to pH 4.0 with 5 M HCl prior to heat sterilisation. Unless otherwise stated, starter cultures comprised 10 ml YEPD pH 4.0 in 28 ml McCartney bottles, inoculated with the yeast on ceramic beads (Microbank™) and incubated for 48 hours at 25 °C.

Resistance to weak-acid preservatives was determined by the minimum inhibitory concentration (MIC) of each acid to completely inhibit growth as described in Stratford et al. (2013). The MIC was the lowest concentration of preservative at which no growth was detectable at 40 days.

### 2.3 Microtitre method of colony counting

Resistance of individual yeast cells was determined by colony growth in static liquid culture, as fully described by Stratford et al. (2013). Briefly, the numbers of cells in starter cultures of *Z. bailii* in YEPD pH 4.0 were accurately counted by haemocytometer and were inoculated with 15–30 cells/ml final concentration and dispensed into flat-bottomed 96-well microtitre plates at 200 μl/well (maximum 3–6 colonies/well). After 40 days, the total colony number at each sorbic acid concentration was recorded and expressed in proportion to the colony number growing in YEPD pH 4.0 without sorbic acid.

### 2.4 Agar method of colony counting

The effects of weak-acid preservatives are strongly influenced by the acidity of the medium. It was therefore necessary to devise a method for accurate pH adjustment of YEPD agar. Agar degenerates in hot, low pH conditions and it was therefore necessary to autoclave agar at near-neutral pH and then acidify it just before pouring. YEPD medium was prepared without agar (not pH-adjusted) and sorbic acid was added to the defined concentrations. Samples (50 ml) were removed from each medium and were titrated to pH 4.0 with HCl (5 M) to determine the volume of acid needed to adjust each batch to pH 4.0. Agar was then added (16 g/l) to the non-pH adjusted medium, which was then warmed to melt the agar before autoclaving. Bottles of media were then held at 50 °C, before acidification to pH 4.0 with HCl (5 M), and poured into Petri dishes (standard 90 mm). Control tests showed that agar had no effect on pH or buffering. As in the microtitre method, the numbers of cells in *Z. bailii* cultures were accurately counted by haemocytometer and serially diluted in YEPD. One hundred microlitre samples containing 100–300 viable cells were spread onto agar plates and incubated at 25 °C for up to 40 days. Agar plates were bagged to prevent evaporation and colony counts were recorded every 2 days.

### 2.5 Culture age effects on sub-populations of *Z. bailii*

Starter cultures were normally initiated by addition of frozen ceramic beads containing *Z. bailii* cells to 10 ml aliquots of YEPD pH 4.0 in 28 ml McCartney bottles. To obtain more reproducible growth patterns, 10 ml YEPD pH 4.0 starter cultures were inoculated with 10^5^ cells/ml (final) from 5 day-old cultures. Multiple replicates of 10 ml cultures were incubated without shaking (static) at 25 °C. When tested, each static starter culture was vortexed, sampled and then discarded. Alternatively, shaking flask starter cultures comprising 40 ml YEPD pH 4.0 in 100 ml conical flasks were inoculated with 10^5^ cells/ml (final) and agitated at 120 rpm on an orbital shaker at 25 °C.

### 2.6 Glucose determination in starter cultures of *Z. bailii*

YEPD contains 2% w/v glucose (111 mM). It is commonly known that yeast growth in YEPD is glucose-limited. Addition of extra glucose increases the yeast growth yield. The time at which glucose was depleted was determined by measurement of the glucose concentration in the media during yeast growth in static and shaking cultures. Samples were adjusted to pH 6.6 and glucose was determined quantitatively using ACCU-CHEK-Aviva blood glucose test strips (Roche Diagnostics GmbH, Mannheim, Germany).

### 2.7 Lag phase of *Z. bailii* in sorbic acid

Detailed assessment of the prolonged lag phase of *Z. bailii* in sorbic acid was carried out in 10 replicate 400 ml cultures of YEPD pH 4.0 containing 6 mM sorbic acid in 1 litre conical flasks. Flasks were inoculated with 5000 cells/ml and agitated at 120 rpm at 25 °C. Flasks were sampled for growth by optical density (OD-600 nm), by plating out onto YEPD agar and YEPD 6 mM sorbic acid agar, by microtitre plates in YEPD and YEPD 6 mM sorbic acid, and by microscopic haemocytometer counting. Early stage samples were centrifuged (2000 ×*g* 3 min) to concentrate (× 1000) the cell count.

### 2.8 Measuring resistance of sub-populations of *Z. bailii*

Tests of population heterogeneity showed very few *Z. bailii* colonies growing at high concentrations of sorbic acid. Single colonies of cells growing in 6 mM sorbic acid after 14 days were mixed in their microtitre plate wells, and cells were counted by haemocytometer. Cultures were then serially-diluted to 10^4^ cells/ml in YEPD pH 4.0 containing 6 mM sorbic acid and used to inoculate microtitre dishes as described previously over the range 0–13 mM sorbic acid. These experiments were repeated using benzoic acid 0–18 mM and acetic acid 0–800 mM, using *Z. bailii* in YEPD corrected to pH 4.0.

### 2.9 Reversion of resistant sub-populations of *Z. bailii*

As previously described, single colonies of *Z. bailii* growing in microtitre plates in 6 mM sorbic acid after 14 days were mixed and the cells were counted by haemocytometer. Each was then inoculated into 10 ml of sorbic-acid-free YEPD pH 4.0 at 10^4^ cells/ml. At each time point, 0–10 hours, samples were inoculated into 20 ml aliquots of YEPD containing sorbic acid (0–13 mM) and dispensed into microtitre plates at 200 μl/well. Plates were sealed, lidded, double-bagged to prevent evaporation, and incubated at 25 °C for 40 days.

### 2.10 Measurement of cellular internal pH by flow cytometry

The method used for determination of cellular internal pH by flow cytometry was a modification of the method described in Stratford et al. (2009). *Z. bailii* was cultured in 40 ml YEPD pH 4.0 in 100 ml conical flasks shaken for 12–16 hours at 120 rpm and 25 °C. Exponentially-growing yeast cells were obtained from shake flasks at OD_600_ 0.15–0.2 following an 11-fold dilution in water. Sub-populations of cells from 6 mM sorbic acid medium in microtitre plates were inoculated into 40 ml of the same medium and shaken for 5 days. CFDASE (carboxyfluoresceindiacetate succimidyl ester) was added to 1 ml samples of yeast in the growth medium at 10 μg/ml final concentration and cells were incubated at 25 °C for 30 min for uptake of the CFDASE. The internal pH of populations of individual fluorescent cells was determined from the linear ratio of the 575 nm (largely pH-independent) and 525 nm (pH-dependent) emission signals. Calibration was carried out using cells of defined intracellular pH, permeated using 2 mM 2, 4-dinitrophenol in 0.7 M acetate–100 mM succinate–100 mM KH_2_PO_4_ buffer, and 100 μM nigericin in the same permeating buffer.

## 3 Results

### 3.1 The effect of culture age on population heterogeneity in *Z. bailii*

**Fig. 1:**
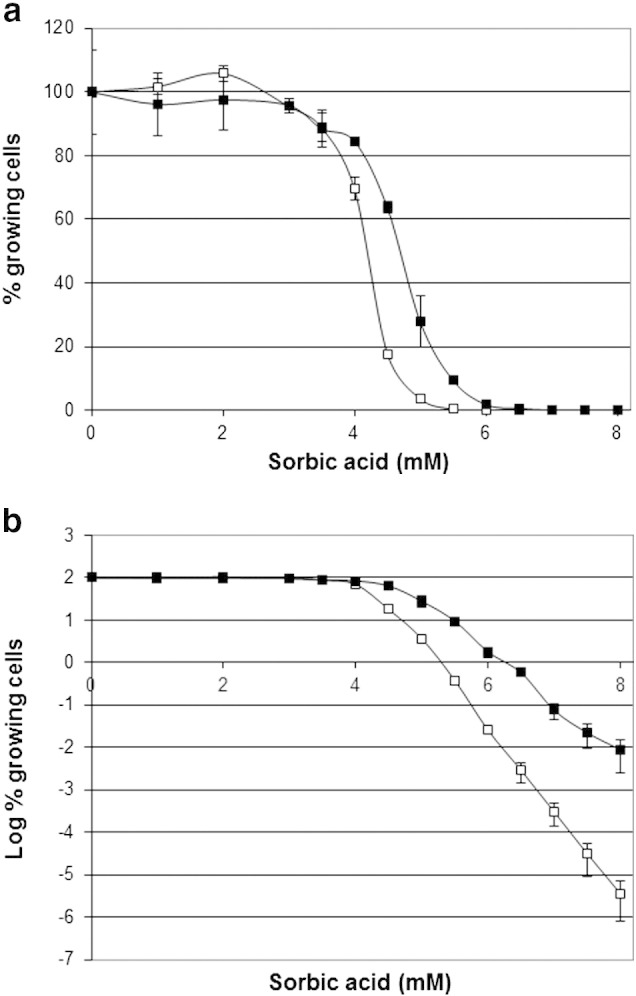
The difference in sorbic acid resistance between exponential- and stationary-phase cultures. a. The proportion of growing cells within populations of *Z. bailii* NCYC 1766 exposed to sorbic-acid, was measured by counting CFU in YEPD pH 4.0 (microtitre method) over 18 days at 25 °C. Starter cultures were exponentially growing (open squares) or stationary phase (closed squares) cells. Data represent the mean and standard deviations from experiments carried out in triplicate. The slope of the “tail” when the proportion of growing cells is reduced from 100% to zero provides a measure of heterogeneity within the population (Holland et al., 2013; Sumner et al., 2003). b. Data plotted as log-base-10 percentages of growing cells in the population.

The extreme extent of resistance of *Z. bailii* to weak-acid preservatives was shown to be due to the presence of an extended “tail” of highly resistant cells. The proportion of the resistant sub-population declined with increased weak-acid concentration, resulting in survival of a few cells in high concentrations of sorbic acid (Fig. 1a). It was found that the proportion of resistant cells in the population varied considerably between different experiments. The cause of this variation was identified as the age of the starter culture. A detailed analysis of population diversity in sorbic acid resistance (Fig. 1a) showed a considerable difference between exponentially-growing cells (38-hour starter culture) and a post-exponential, stationary-phase culture (52-hour). It was found that, in both cultures, the bulk cell population was fully resistant to 3 mM sorbic acid but at higher concentrations of sorbic acid, a larger proportion of the stationary phase cells (compared to cells in exponentially-growing populations) was able to grow. The slope of the “tail” when the proportion of growing cells was reduced from 100% to zero has been shown to provide a measure of heterogeneity within the population (Holland et al., 2013; Sumner et al., 2003). In this instance, the heterogeneity of the stationary phase population is clearly higher than that of the exponential population. Plotting the data using log base 10 (Fig. 1b) shows that the discrimination between the populations at different growth stages increases with the concentration of sorbic acid. The decline in surviving and growing cells is very close to log-linear (linear on a log plot), very closely resembling cell death plots caused by heat. In heat-kill plots the *D*-value is a measure of the time at a particular temperature for a 10-fold (1-log) kill. In the sorbic acid plots above 4.5 mM, the 1 log-reduction (*D*-value) was ~ 0.53 mM sorbic acid in exponential cultures and ~ 0.80 mM sorbic acid for stationary phase cells (Fig. 1b).

The slopes of these plots demonstrate that the difference between exponential- and stationary-phase cells rises with increased concentrations of sorbic acid. At 6 mM sorbic acid the resistant sub-population proportion was 1 cell in 4000 in the late exponential phase, and 1 cell in 56 in the stationary phase, a 70-fold change. At 8 mM the resistant population was 1 cell in 28 million exponential cells and 1 in 11,000 stationary cells, a 2500-fold increase. These experiments were repeated using microtitre and agar methods of colony counting, with very similar results (data not shown).

**Fig. 2:**
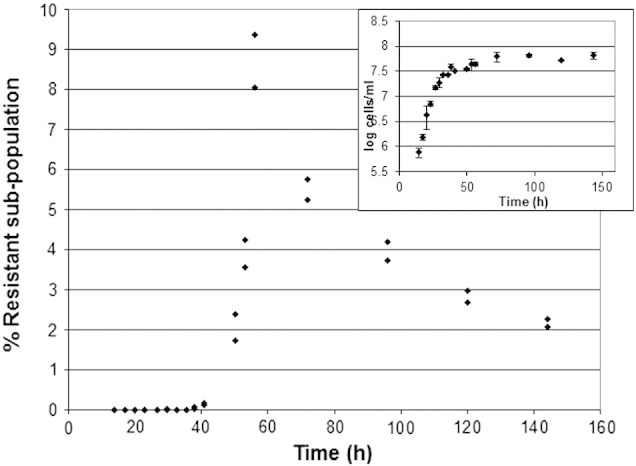
Rise in the resistant sub-population during static culture, after the end of exponential growth. Percentage of the resistant sub-population of *Z. bailii* in static 10 ml cultures of YEPD pH 4.0 at 25 °C, able to grow in 6 mM sorbic acid, was determined by the microtitre method after 14 days. Each point indicates a separate culture. Insert: Growth curve (log10 plot) of *Z. bailii* in static culture.

**Fig. 3:**
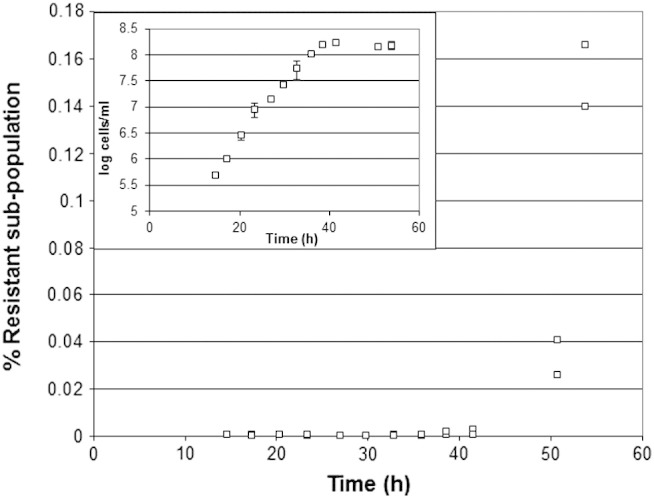
Rise in the resistant sub-population during shaking culture, after the end of exponential growth. Percentage of the resistant sub-population of *Z. bailii* cells in shaking 40 ml cultures of YEPD pH 4.0 at 25 °C, able to grow in 6 mM sorbic acid, determined by the microtitre method after 14 days. Insert: Growth curve (log10 plot) of *Z. bailii* in shaking culture.

Detailed analysis of the resistant sub-population in starter cultures was carried out (Figs. 2 and 3) at progressively later stages through exponential growth and into stationary phase. In static cultures, it was found that the exponential growth phase ended shortly after 38 hours (Fig. 2), although growth continued at a progressively slower rate to beyond 50 hours. Examination of the proportion of the 6 mM sorbic acid resistant sub-population present confirmed that the level of resistant cells during exponential growth was exceptionally low. The average proportion of the sub-population during exponential growth (17 hours–36 hours) was close to 1 cell in a population of 100,000, resistant to 6 mM sorbic acid. By 41 hours, this proportion began to rise to 1 in 700, increasing to a peak at 56 hours, with the sub-population at 1 cell in 12 (8–9% of the total population). This rise in the sub-population amounts to a rise of greater than 8000-fold (Fig. 2) while the total population increased by ~ 2-fold. The time difference between the end of exponential growth and the final peak in the resistant sub-population was 18–20 hours. It was calculated that the 8000-fold rise is not likely to be caused by growth of the resistant sub-population. Such a population rise would require 13 doublings in population, and as the optimal exponential doubling time of *Z. bailii* is marginally longer than 2 hours, 13 doublings would require in excess of 26 hours. It is therefore possible that this rise in the resistant sub-population was due to altered gene expression in a proportion of the bulk population. After 60 hours, when the growth of the total population had effectively ceased, the proportion of the resistant sub-population was observed to fall slightly but progressively, reaching ~ 2% by 144 hours (Fig. 2).

Microscopic examination of cells in exponential phase showed the population to be fairly homogeneous, with ovoid budding cells. By 54 hours, the population appeared more heterogeneous. Some cells remained ovoid but many were thinner, and more elongated, with some budding cells remaining.

These experiments were repeated using shaking flasks (120 rpm) as starter cultures. In both shaking and static cultures, the proportions of resistant cells rose dramatically after the end of the periods of exponential growth (Fig. 3). Shaking exponential growth ceased at close to 38–40 hours (Fig. 3) with little further growth detected. The end of exponential growth was found to be coincidental with glucose limitation in YEPD. Glucose depletion in shake flask cultures occurred close to 39 hours. The resistant sub-population increased in proportion from this time rising substantially by 54 hours (Fig. 3), approximately 16 hours after the end of exponential growth. In static cultures, glucose depletion was later at 66–70 hours.

### 3.2 The effect of population heterogeneity on lag-phase adaptation in *Z. bailii*

It is generally accepted that weak-acid preservatives, including sorbic acid, cause very prolonged lag phases in yeast growth. Given that only a small proportion of the bulk population appears to be resistant to the weak-acid when tested using agar or microtitre methods, it was hypothesised that the extended lag phase could represent the time required for the resistant sub-population to grow and replace the non-resistant population. This was tested in detail measuring cell growth by optical density, haemocytometer counting, and viable colony counting on microtitre plates and on agar media in Petri dishes (standard 90 mm).

**Fig. 4:**
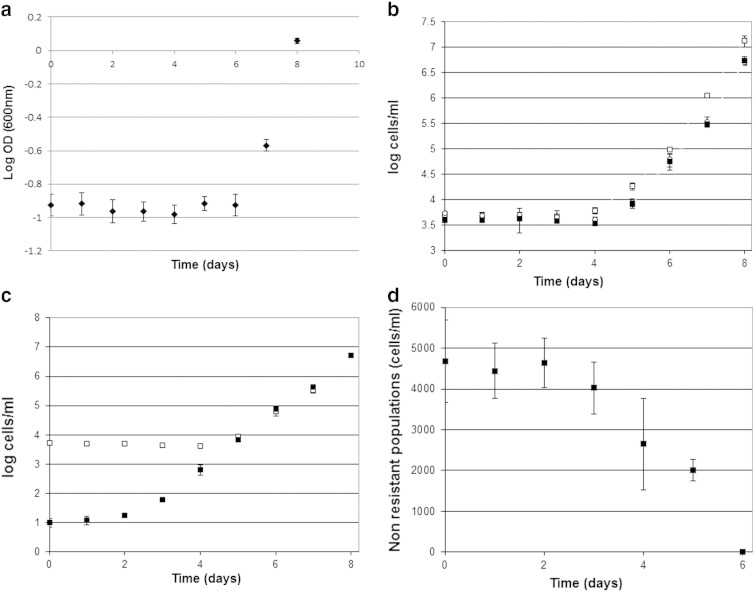
Log10 plot of optical density (OD 600 nm) measurement of lag phase in *Z. bailii* in shake flasks of YEPD pH 4.0 containing 6 mM sorbic acid at 25 °C. a. Data represent the mean and standard deviations of data from experiments carried out in triplicate. b. Lag phase in *Z. bailii* (log10 plot) measured by haemocytometer (open squares), CFU on agar plates (open circles) or microtitre plates (closed squares) in shake flasks of YEPD pH 4.0 containing 6 mM sorbic acid at 25 °C. Data represent the mean and standard deviations of data from experiments carried out in triplicate. c. Log10 plot of lag phase of *Z. bailii* in the total population and the resistant sub-population growing in shake flasks of YEPD pH 4.0 containing 6 mM sorbic acid at 25 °C, measured by CFU on agar containing 6 mM sorbic acid (closed squares) or 0 sorbic acid (open squares). Data represent the mean and standard deviations of data from experiments carried out in triplicate. d. Death of the bulk population of *Z. bailii* during lag phase in shake flasks of YEPD pH 4.0 containing 6 mM sorbic acid at 25 °C. Data comprise the subtraction of the resistant sub-population from the total population, averaged using the agar and microtitre data and representing the mean and standard deviations of experiments carried out in triplicate.

Data from optical density (600 nm) measurement showed an apparent lag phase of 6 days before growth was detected (Fig. 4a) in shake flasks containing YEPD pH 4.0 with 6 mM sorbic acid (by comparison, the lag phase in YEPD lacking sorbic acid was ~ 1 hour). Measurement by colony forming units (CFU) on agar or microtitre plates showed a lag phase of 4 days before growth was detected at 5 days (Fig. 4b). However, data on the growth of the resistant sub-population showed a lag phase of only 2 days, followed by a slow exponential growth of the sub-population (Fig. 4c) with a doubling time of ~ 8 hours. It was notable that the exponential doubling time of the sub-population was nearly identical with that of the total population after 5 days. Repeating the experiment using agar detection of the sub-population gave near identical results. The bulk population of non-resistant cells was determined by subtraction of the resistant sub-population from the total population. These results (Fig. 4d) showed that the bulk population cells appeared in stasis for the first 3 days (no growth, no death), before cell death was initiated by day 4, and was largely complete by day 6. This suggests that the sensitive population progressively died between day 3 and day 6, being progressively replaced by the resistant sub-population.

### 3.3 Adaptation (enhanced MIC) in *Z. bailii* caused by pre-growth in 6 mM sorbic acid

The concentration of sorbic acid required at pH 4.0 to inhibit the growth of an inoculum of 10,000 *Z. bailii* cells typically averaged above 7 mM. It was found that this inhibitory concentration (MIC) was closer to 6.5 mM using exponential cells, and 8.5 mM using stationary phase cells. However, if *Z. bailii* was cultured in 6 mM sorbic acid for 1 week (shaking culture), to allow population replacement with the resistant sub-population, all cells in the population were now able to grow at 6 mM sorbic acid and tolerated higher concentrations of sorbic acid. MIC tests of resistant 10^4^ cell sub-populations pre-grown in 6 mM sorbic acid, compared with normal populations, altered the MIC from 8.5 mM to 12.5 mM sorbic acid, and also from 9 mM to 17 mM benzoic acid and from 500 mM acetic acid to 700 mM acetic acid (data not shown). The growth rate of colonies (both agar and microtitre methods) progressively slowed at higher concentrations of weak acid preservative. Colony growth (from large inocula) in 13 mM sorbic acid required 40 days to achieve a diameter of 1 mm. Similar results were obtained with *Z. bailii* pre-grown in 6 mM sorbic acid in static culture, or in microtitre plates for 14 days.

### 3.4 Stress removal causes loss of resistance in *Z. bailii*

**Fig. 5:**
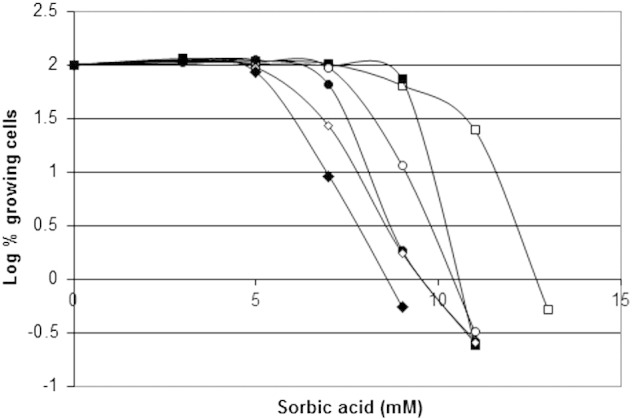
Loss of sorbic acid resistance (log10 plot) after removal of resistant *Z. bailii* from sorbic acid. *Z. bailii* resistant sub-populations were pre-cultured for 14 days in static culture YEPD 6 mM sorbic acid at 25 °C, and transferred to static culture YEPD (no sorbic acid). Subsequently, populations were sampled (time 0 open squares), after 2 hours (closed squares), 4 hours (open circles), 6 hours (closed circles) 8 hours (open diamonds) and 10 hours (closed diamonds). Colonies were then counted after growth on YEPD broth (microtitre method) containing the indicated concentrations of sorbic acid. Sample populations of > 8000 cells were grown in 96-well microtitre plates, at 300–600 cells/plate.

Examination of the range of resistance in sub-populations pre-grown in 6 mM sorbic acid showed that all cells were resistant to 8 mM sorbic acid with a logarithmically declining proportion of cells capable of growth in medium containing 13 mM sorbic acid (Fig. 5). It had previously been shown that placing resistant sub-populations overnight into non-selective medium (YEPD) caused reversion back to the normal population. This was examined in detail, sampling the population for heterogeneity every 2 hours. The results confirmed a rapid loss of the ability to grow in the highest levels of sorbic acid (Fig. 5). After 2 hours in non-selective medium, the small population able to grow in the extreme levels of sorbic acid had declined substantially, with a large effect on resistance to 11 mM sorbic acid, but little effect at 9 mM sorbic acid. Similar effects occurred after 4 hours, with a large reduction in the population able to grow in 9 mM sorbic acid but with little effect at 7 mM (Fig. 5). The rapidity of change within 2 hours (1 generation time at maximum growth rate) suggests that the rapid reversion to the normal population may possibly be caused by alterations in gene expression, contributed to by the rapid growth rate of the reverted cells, and not by replacement of the resistant population by the sensitive one.

### 3.5 Population diversity in *Z. bailii* under sorbic acid stress

**Fig. 6:**
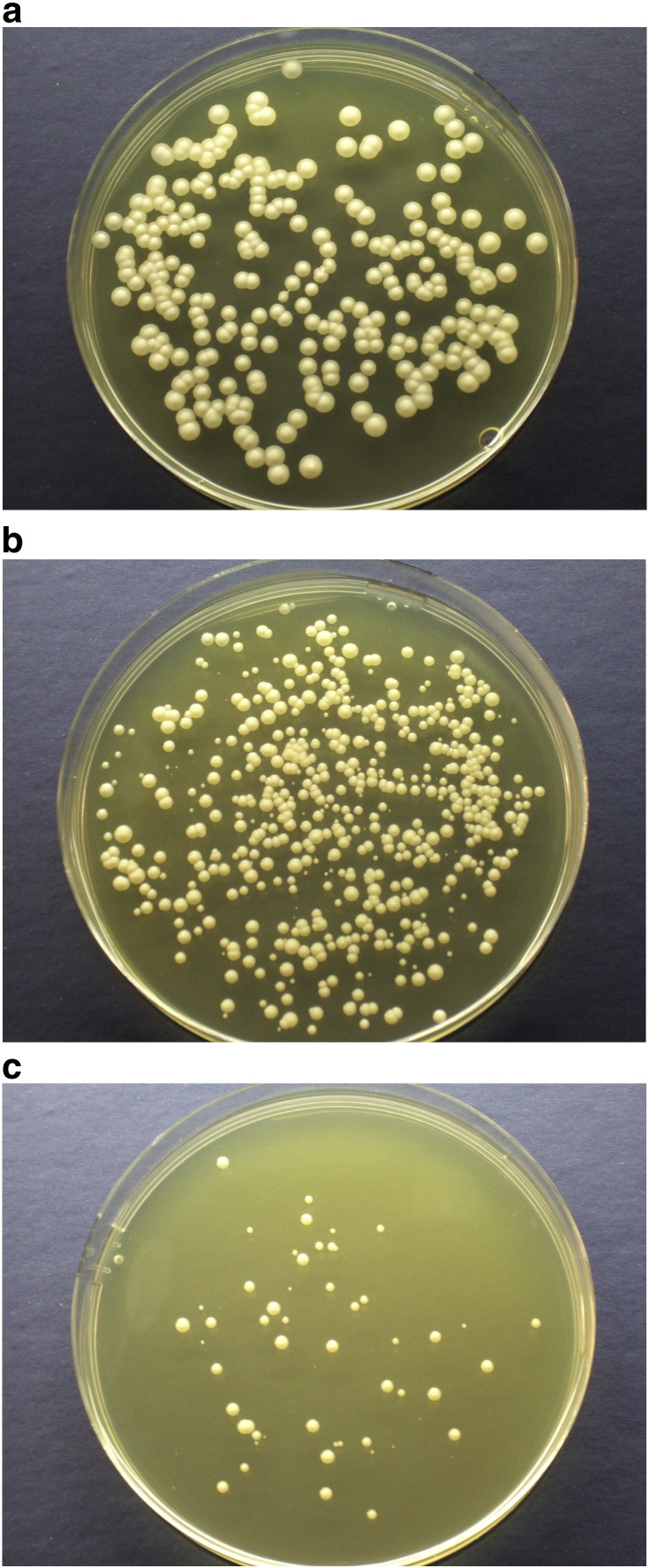
Colony morphology of *Z. bailii*. a. Homogeneous morphology of colonies of *Z. bailii* on YEPD pH 4 agar after 6 days growth at 25 °C. b. Diversity in colony size of *Z. bailii* under 4.5 mM sorbic acid stress (YEPD pH 4 4.5 mM sorbic acid agar after 9 days growth at 25 °C). c. Diversity in colony size of *Z. bailii* under 6.5 mM sorbic acid stress (YEPD pH 4 6.5 mM sorbic acid agar after 14 days growth at 25 °C).

Thus far, the diversity of *Z. bailii* cell populations was seen to change markedly a few hours after the end of exponential growth, with respect to sorbic acid resistance. In the presence of sorbic acid, the bulk population appeared to progressively die and be replaced by the slow-growing sub-population. There was no evidence of any of the bulk population becoming resistant, once the sorbic acid stress had begun. However, there was evidence of further population heterogeneity development in the sub-population that had survived the sorbic acid stress. The normal non-stressed population, when assessed using either agar or microtitre methods, showed colony size to be very homogeneous with less than 0.2% of colonies being unusually small (Fig. 6a), both during exponential (36 hours) and stationary phases (55 hours), irrespective of the higher proportion of sorbic acid-resistant sub-population in stationary phase cultures. However, in the presence of sorbic acid, colonies of a much smaller size than the average population became apparent and these increased in relative frequency as the concentration of sorbic acid increased. In 3 mM sorbic acid where all cells remain viable, colony size diversity was observed. In 4.5 mM sorbic acid, small colonies comprised ~ 10% of the population, reflecting a slow growth rate (Fig. 6b), while in 6.5 mM sorbic acid, this rose to ~ 25% (Fig. 6c). When the small colonies that had formed under sorbic-acid stress were restreaked onto YEPD, all colonies were found to have reverted to the large size of control colonies, and grew rapidly.

**Fig. 7:**
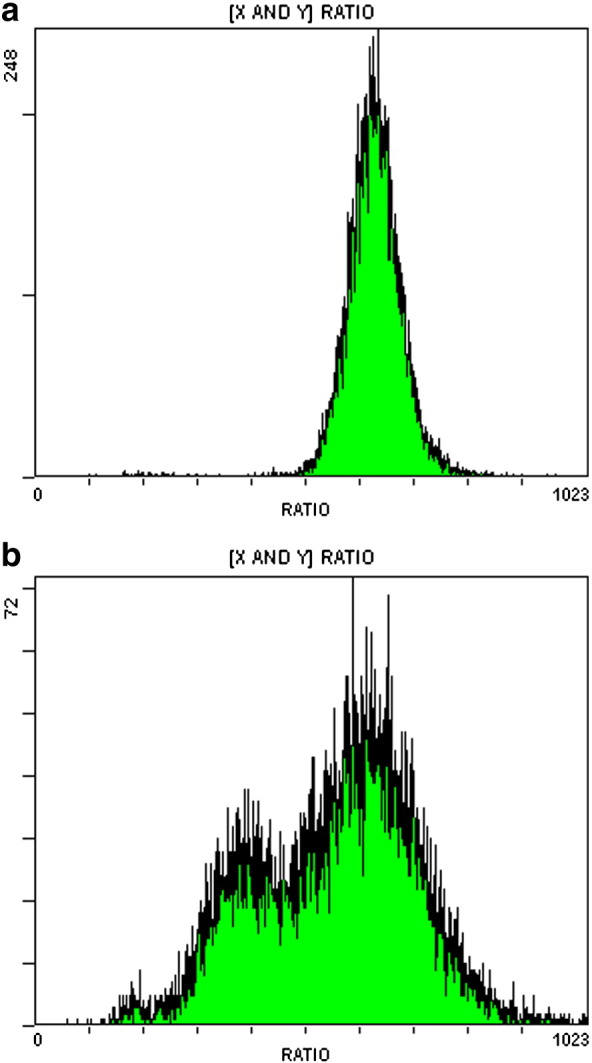
Flow cytometer plots of frequency distribution of the internal pH of individual cells from exponentially-growing cell populations of *Z. bailii* in YEPD pH 4.0 at 25 °C. The *x*-axis shows the cell internal pH using the ratio of the 575 nm (largely pH-independent) and 525 nm (pH-dependent) CFDASE emission signals (high ratio = high pH). The *y*-axis shows the frequency count. a. A normal population of cells from exponential culture in YEPD, mean internal pH 6.2. b. The resistant sub-population of cells from exponential culture (100% resistant cells) growing in YEPD containing 6 mM sorbic acid, mean internal pH 5.6.

High-level population heterogeneity in the presence of sorbic acid was also reflected in tests on the internal pH of the normal exponentially-growing population compared with the exponentially-growing resistant sub-population growing in 6 mM sorbic acid. The control population had a mean internal pH of 6.2 with a narrow distribution (Fig. 7a). The sub-population (100% resistant cells) had a lower mean internal pH (5.6) but with a very wide pH distribution of cells (Fig. 7b). Some cells had an internal pH comparable to the normal population, while others had very low internal pH.

## 4 Discussion

The results in this paper corroborate earlier studies (Stratford et al., 2013) that showed population heterogeneity in isogenic cell populations of *Z. bailii* to be the principal cause of extreme resistance in this yeast species to weak acid preservatives. Population heterogeneity can in theory be altered by the fraction of the population in a phenotype or by the numbers of different phenotypes present, or both. There are also several possible causes of population heterogeneity. This could be due to variations in expression of one, or several genes via regulatory pathways, or possibly due to alteration in the properties of cellular components, such as proteins, due to non-genetic, stochastic activation–deactivation e.g. phosphorylation or alteration in conformation. Such an alteration could be perpetuated by epigenetic inheritance (Veening et al., 2008), a non-genetic feedback loop or via self-generation as in yeast prions (Tuite, 1994). However, in the current study, the progressive changing “tail” of population resistance would appear to be more likely due to progressive variation in gene expression, rather than epigenetic bistability, predicted to result in two distinct populations.

The resistant sub-population to sorbic acid is extremely rare in exponentially-growing cultures. However, after the end of exponential growth, there is an explosive rise in the level of the resistant sub-population. The timing of this rise appears to be consistent with a response to starvation stress caused by the depletion of glucose. Previous studies (Holland et al., 2013) have indicated increases in yeast population heterogeneity within stressful environments. Several yeast species isolated from natural environments containing high levels of toxic heavy metals or sulphur dioxide were found to have enhanced levels of diversity within the genetically-uniform populations. It is thought that elevated heterogeneity in stressed populations increases the likelihood of at least a few cells surviving. This includes heat-kill survival in yeast, with heterogeneity proposed to be a method of “bet hedging” (Levy et al., 2012). It is likely that the dynamic nature of phenotypic (versus genotypic) heterogeneity is best suited to priming sub-populations for future stressful perturbations that may arise. Therefore, to be most effective, population heterogeneity should be multi-dimensional, to cover the great variety of causes of cell death, physical stresses such as heat, radiation or pressure, chemical toxicity of enormous variety, or environmental effects such as starvation, or oxidative stress. The results shown here are consistent with this proposal. In addition to the increase in the weak-acid resistant sub-population after exponential growth, there were changes in microscopic cell morphology. Sorbic acid stress caused death to the bulk population of yeast cells, but also led to a notable variation in colony size of the survivors. Further evidence of the extent of stress-related population heterogeneity was seen in MIC tests in static cultures. Preliminary data showed only a few resistant cells (tail) at the MIC from a variety of yeast species, stressed by salt, glucose, Velcorin, H_2_O_2_, EDTA, citrate, heat, nickel, copper, low or high pH (M. Stratford, unpublished data). It is also widely known in the food industry that exponentially-growing microbes are more sensitive to stresses than those in stationary phase. This includes heat, osmotic and oxidative stress in bacteria (Zambrano and Kolter, 1995) and in yeast (Werner-Washburne et al., 1996). This raises the possibility that population heterogeneity is a common resistance mechanism to a variety of stresses, by many species, and that the proportions of several resistance phenotypes are rare during exponential growth but expands during stresses such as stationary phase.

While population heterogeneity effects on stress resistance have been recently recognised, historically there has been evidence of this effect that was not recognised at the time. During pasteurisation in the food industry, heat kill of microbes has long been recognised as variable. Under heat stress, populations die in a reverse exponential fashion, giving linear log-plots over time and providing *D*-values for heat kill; the time required to kill 90% of the population (Put et al., 1976). In addition, a resistant “tail” in the population was frequently found, giving rise to animated disputes as to the cause. It was commonly regarded as an artefact caused by a few cells not being properly exposed to the heat. Recent studies (Levy et al., 2012) have shown that heat-resistant cells of the yeast *Saccharomyces cerevisiae* were slow growing and resistance could be related to expression of the *TSL1* gene (involved in trehalose-synthesis regulation). It is known that the presence of trehalose confers structural stability on proteins and lipids (Iwahashi et al., 1995; Sillje et al., 1999) and a role for trehalose in stress resistance has been established (Londesborough and Vuorio, 1991). Bacterial cells also use population heterogeneity to escape elimination by antibiotics. Similarly to the sorbic acid resistant sub-population described in the current paper, slow-growing “persister cells” form a small fraction of cells in exponential populations, which rises up to 1% in stationary phase or in biofilms (Lewis, 2008; Wood et al., 2013). Persisters are phenotypically dormant (unlike yeast sub-populations) and do not grow in the presence of antibiotics, but survive the treatment by shutting down metabolism. Diversity in cell gene expression and physiology may also be implicated in the observations that many species of pathogenic bacteria have a minimum infectious dose. *Escherichia coli* 0157:H7 is unusually dangerous with an infectious dose of a dozen cells (Butler, 1996), *Salmonella typhimurium* generally requires ingestion of ~ 1000 cells to cause illness (Blaser and Newman, 1982) and *Campylobacter jejuni* requires 800–1,000,000 cells (Black et al., 1988). Research on population heterogeneity makes it possible that these figures represent the statistical probability of finding one pathogenic cell within this size of population, or one cell able to survive the acidity of the stomach.

It is generally accepted that treatment of microbial cells with a stress agent will result in a change in gene expression, an adaptive response to enable survival and growth of the cells. Evidence is presented here that the lag phase of yeast treated with a weak-acid preservative does not represent an adaptive response but, instead, the time required for replacement of the bulk population by a slow-growing resistant sub-population. The bulk population shows no obvious sign of any adaptive response but dies slowly over 6 days. Why is there no adaptation? The data on sorbic acid cell death show great similarity to heat-kill with a log-linear death response. Heat kill is rapid, and it is understandable that there is no time for alteration in physiology due to gene expression. Sorbic acid kills slowly in comparison, and it would be expected that there would be sufficient time for a cell response, as has been shown with low concentrations of sorbic acid used against *S. cerevisiae* (Schuller et al., 2004). However, sorbic acid behaves as if it is killing the cells quickly and as if it were blocking cell responses. The data suggest that sorbic acid toxicity prevents a survival response in the bulk population; these cells are committed to death (albeit slowly) and only the cells in the surviving sub-population show increased diversity in response to the sorbic acid stress.

The evolutionary implications of these results may also be significant. The Neo-Darwinian theory of evolution proposes that natural selection eliminates a large proportion of populations, resulting in “survival of the fittest.” Such selective pressures on genetically-mixed populations would result in loss of genetic diversity. Population diversity is important for survival, given a large element of chance in survival of extinction events (Gould, 1990). The organism may evolve through mutation to become characterised by increased heterogeneity; the most likely explanation being evolution of “noisier” (less tightly regulated) expression of certain genes (Holland et al., 2013). It is already known that sequence elements in gene promoters can determine how noisy their expression is. The evidence presented here shows that the proportions of cells occupying different phenotypic states can change under stress. This may enable survival of a few individuals under selection pressure, and yet without excessive loss of phenotypic (or genetic) diversity as protection from other, later selective pressures.

In a summary of results, *Z. bailii* is the most preservative resistant species of all fungi (James and Stratford, 2011; Thomas and Davenport, 1985) and it was shown here that cell survival was caused by a resistant sub-population. Increased heterogeneity is generated under stressed circumstances. Weak-acid preservatives at high concentrations will kill the vast majority of the population giving no possibility for survival by adaptive response. During lag phase, the population is replaced by the slow growing resistant sub-population. Once the stress is removed, the population rapidly reverts to normal, enabling rapid growth in the surviving cells.
